# Remarks on Lucorrhœa

**Published:** 1871-03

**Authors:** Jas. L. Brown

**Affiliations:** New York


### REMARKS ON LEUCORRHŒA.

BY DR. JAS. L. BROWN, OF NEW YORK.

Mrs. M., æt. 31; has five children, youngest 9 years old. For three years has had a pain in the back, and irritation of the bladder, with constant discharge from the vagina. This discharge was watery and offensive, and sometimes tinged with blood. Upon any exertion this runs from the patient in a stream. Has not menstruated in three years.

Dr. Brown said the history of the case was exactly what would lead us to suspect epithelioma; but on making a vaginal examination the cervix is found to be normal. Flowing from the os was a stream of pus. On introduction of the sound the patient complained of pain in the fundus. The intra-uterine measurement was 2½ inches. The diagnosis of the case is corporeal endometritis, the first of the kind that has been presented to the class this season.

Dr. B. said that he would take for his text leucorrhœa, a symptom the most common of all diseases of women, and would consider this symptom as a disease.

There are three main sources of it: vaginitis, *cervical* and *corporeal endometritis;* but it is also derived from every form of uterine derangement, malposition, out-growths, etc.

The question often arises how much of a discharge constitutes leucorrhœa? This has been settled conventionally, by deciding it to be such when the linen is soiled by it.

The diagnosis of cervical from corporeal endometritis is exceedingly difficult, but when a limpid slimy mucous is discovered coming from the os, endocervicitis in all probability exists. And when the discharge is opaque and thick, or even purulent, we suspect endometritis.

Inflammation of the cervix offers the best hope of cure; three or four months sufficing. The prognosis of endometritis, on the other hand, is by no means so favorable. Scanzoni states that he has never cured a single case of chronic leucorrhœa.

*Treatment.*—Inasmuch as leucorrhœa is a symptom of debility, tonics are especially indicated.

Plain water injections morning and evening are of service. Half a gallon to be used at a time. If there appears to be endocervicitis, a solution of nitrate of silver (xxx gr. ℥ i.) may be applied once a week. I have found this to be more satisfactory than anything else.

In respect to the case before us, there is very little hope of curing her completely.

First—Tonics will be ordered; a piece of cotton will be saturated with the solution of nitrate of silver, and introduced into the cavity of the uterus, and within twenty-four hours the organ will have expelled it.

Gynæcologists, in this city, are at present discussing the use of intra-uterine injections. The majority are much opposed to them, many thinking that they have frequently caused the death of patients.—*Med. and Surg. Reporter.*

